# Cerebral Blood Flow and Blood–Brain Barrier Water Exchange in Major Depressive Disorder: Evidence from Diffusion-Prepared Arterial Spin Labelling MRI

**DOI:** 10.3390/brainsci16010027

**Published:** 2025-12-25

**Authors:** Simonas Jesmanas, Eglė Milašauskienė, Julius Burkauskas, Vilmantė Borutaitė, Kristina Škėmienė, Virginija Adomaitienė, Brigita Gradauskienė, Saulius Lukoševičius, Rymantė Gleiznienė, Guy C. Brown, Vesta Steiblienė

**Affiliations:** 1 Department of Radiology, Lithuanian University of Health Sciences, LT-50009 Kaunas, Lithuania; saulius.lukosevicius@lsmu.lt (S.L.); rymante.gleizniene@lsmu.lt (R.G.); 2 Laboratory of Behavioral Medicine, Neuroscience Institute, Lithuanian University of Health Sciences, LT-50161 Kaunas, Lithuania; egle.milasauskiene@lsmu.lt (E.M.); julius.burkauskas@lsmu.lt (J.B.); vesta.steibliene@lsmu.lt (V.S.); 3 Laboratory of Biochemistry, Neuroscience Institute, Lithuanian University of Health Sciences, LT-50161 Kaunas, Lithuania; vilmante.borutaite@lsmu.lt (V.B.); kristina.skemiene@lsmu.lt (K.Š.); 4 Department of Psychiatry, Lithuanian University of Health Sciences, LT-50161 Kaunas, Lithuania; virginija.adomaitiene@lsmu.lt; 5 Department of Immunology and Allergology, Lithuanian University of Health Sciences, LT-50009 Kaunas, Lithuania; brigita.gradauskiene@lsmu.lt; 6 Department of Biochemistry, University of Cambridge, Cambridge CB2 1QW, UK; gcb3@cam.ac.uk

**Keywords:** major depressive disorder, blood–brain barrier, water permeability, cerebral blood flow, inflammation

## Abstract

**Background:** Diffusion-prepared pseudo-continuous arterial spin labelling (DP-pCASL) can quantify the cerebral blood flow (CBF) and the water exchange rate (kw) across the blood–brain barrier (BBB). Little is known about the BBB water exchange in major depressive disorder (MDD). **Objective:** We aimed to explore the associations between kw, CBF, peripheral inflammation, and MDD. **Methods:** Using DP-pCASL, we measured the global and selected regional kw and CBF together with blood plasma levels of lipopolysaccharide (LPS) and inflammatory cytokines in 85 patients with MDD and 51 controls. **Results:** The global CBF was significantly lower in MDD patients compared with controls (means of 51 and 57 mL/100 g/min, respectively; *p* = 0.006), with similar reductions found in the dorsolateral and ventromedial prefrontal, anterior, and posterior cingulate regions, while no differences were found in the amygdala and the isthmic cingulate. There were no differences in the kw between groups globally (means of 128 min^−1^; *p* = 0.958) and in the studied regions. Among MDD patients, the kw was weakly correlated with the MADRS scores (r = 0.231, *p* = 0.034). There were no associations between kw, CBF, and inflammatory markers (LPS, IL-6, IL-10, TNF-α, IFN-γ). Logistic regression showed that a combination of the regional CBF < 59.22 mL/100 g/min together with LPS > 143.58 pg/mL and/or IL-10 > 0 pg/mL distinguished MDD patients from controls with a moderate accuracy of 83.1% (sensitivity = 94.1%, specificity = 64.7%, AUC = 0.876). **Conclusions:** DP-pCASL imaging confirmed previous findings of reduced CBF in MDD, which together with LPS and IL-10 concentrations were independent significant predictors of MDD. However, no changes in the BBB water exchange were found, suggesting that it may not be as significant as CBF in MDD pathophysiology.

## 1. Introduction

Major depressive disorder (MDD) is a common affective disorder with numerous hypotheses aiming to explain its pathophysiology, including the inflammatory hypothesis [1]. Dysfunction of the blood–brain barrier (BBB) is known to occur in association with MDD and other psychiatric disorders [2,3]. The BBB protects the brain from endogenous and exogenous neurotoxins [4]. Systemic inflammation can induce non-disruptive (functional) and disruptive (anatomical) changes in the BBB [5]. Transport across the BBB can occur via passive diffusion, active efflux, carrier-mediated transport, transcytosis, and cell diapedesis [4]. Thus, different mechanisms of BBB disruption are possible. Increased BBB water transport can happen not only through reduced tight junction (TJ) integrity, but through aquaporin-4 (AQP4) channels expressed on astrocyte end feet membranes [6]. Increased AQP4 expression (due to any pathophysiological process) would increase water permeability across the BBB, but not permeability to larger molecules, such as gadolinium-based contrast agents (GBCAs). AQP4 channels are also an important part of the glymphatic system which allows for the clearance of metabolic waste products from the brain [7,8]. Glymphatic system dysfunction may induce cytokines and activation of microglial inflammasomes, leading to neuroinflammation, which would further disturb the glymphatic system in a vicious cycle [9].

Dynamic contrast-enhanced magnetic resonance imaging (DCE-MRI) has been used to show increased BBB permeability in patients with MDD when compared with controls, and in untreated patients when compared with treated ones [10]. Another DCE-MRI study revealed increased BBB permeability and its association with depression and anxiety in systemic lupus erythematosus patients [11]. DCE-MRI uses GBCAs which are larger molecules, and their passage through the BBB implies paracellular leakage because of reduced TJ function or expression.

Positron emission tomography (PET) using [^18^F]2-Fluoro-2-deoxy-sorbitol ([^18^F]FDS) was used in a mouse model of endotoxemia and showed that injections of low doses of lipopolysaccharide (LPS) triggered neuroinflammation without causing BBB hyperpermeability, however higher doses of LPS did increase BBB permeability [12]. PET with an 18-kDA translocator protein (TSPO) ligand can be used to assess brain inflammation, because TSPO expression is increased by inflammatory glial cell activation [13,14]. TSPO PET has provided some evidence that peripheral inflammatory cytokines in MDD may be associated with decreased BBB permeability, causing disruption of brain homeostasis and depressive outcomes [15,16].

Recently, new MRI techniques were developed to measure water exchange across the BBB, using contrast-enhanced or non-contrast-enhanced approaches, the latter category including modifications on arterial spin labelling (ASL) [17]. Non-contrast water exchange quantification has the advantage of not requiring the injection of a contrast agent or any other intervention into a subjects’ body. In the absence of BBB breakdown, water exchange across the BBB happens mainly via AQP4 channels, ion channels, and other transmembrane proteins, thus water exchange imaging can assess these components of BBB transport relatively selectively [17].

Diffusion-prepared pseudo-continuous arterial spin labelling (DP-pCASL) is one of the new sequences for measuring water exchange across the BBB [18]. The sequence uses a diffusion weighting (with B = 50 s/mm^2^) to separate the tissue/capillary fraction of the ASL signal and provides a good reproducibility of water exchange measurements (kw) with an intraclass correlation of 0.75 [19]. This DP-pCASL sequence was used to show that the water exchange rate is increased in patients with diabetes and hypercholesterolemia, is associated with cognitive performance (depending on studied regions), CSF amyloid-β 42 in healthy older adults, and is decreased in hereditary cerebral small vessel disease [19,20,21,22].

Importantly, a study comparing DP-pCASL with DCE-MRI showed that water permeability (kw) and gadolinium permeability (ktrans/kgad) were correlated in some regions (particularly white matter, caudate), but not in grey matter or the whole brain; the researchers concluded that the mechanisms underlying the permeability of water and contrast molecules are likely different [23].

To the best of our knowledge, there is very little data on using imaging of water exchange across the BBB in patients with MDD. The only such publication we could find was a conference abstract using intrinsic diffusivity encoding of arterial labelled spins (IDEALS) MRI to investigate 14 MDD patients and 14 subjects, which showed reduced water exchange in the amygdala, anterior cingulate cortex, dorsolateral prefrontal cortex, and hippocampus of MDD patients when compared with controls [24].

DP-pCASL can also be used to measure cerebral blood flow (CBF), providing an opportunity to study two different parameters with one sequence. Indeed, CBF alterations in MDD are well described, with a recent meta-analysis of 15 studies showing significantly reduced CBF of the whole brain in depressed patients, but with heterogeneity between studies [25]. CBF has also been shown to be correlated with peripheral inflammatory markers in MDD [26]. Indeed, multiple inflammatory cytokines are implicated in MDD pathophysiology, including interleukin 6 (IL-6), interleukin 10 (IL-10), interferon-γ (IFN- γ), and tumour necrosis factor α (TNF-α) [27,28,29,30,31,32]. Additionally, endogenous lipopolysaccharide (endotoxin) has been suggested to play a role in neurodegeneration, while its neuroinflammatory role in MDD is currently being investigated [33].

We could not find any studies published in the English language that evaluated MDD patients by using the DP-pCASL sequence. In this study, we aim to address the existing gap in the literature by using DP-pCASL imaging to evaluate changes in the BBB water exchange rate and CBF in patients with MDD to better understand which of these parameters is more prominently altered in MDD, and whether any of them are associated with the blood plasma levels of lipopolysaccharide (LPS) or inflammatory cytokines.

## 2. Materials and Methods

### 2.1. Study Design

A single-centre cross-sectional study was approved by the Kaunas Regional Biomedical Research Ethics Committee (approval No. BE-2-11, 18 January 2022, updated version No. P1-BE-2-11, 17 February 2023). This study was undertaken at the hospital of the Lithuanian University of Health Sciences Kaunas Clinics Psychiatry and Radiology Clinics as part of a broader research project investigating the links between lipopolysaccharide, neuroinflammation, and structural brain changes in major depressive disorder (MDD). The research project was registered on ClinicalTrials.gov (NCT06203015), conducted in accordance with Good Clinical Practice guidelines and the Declaration of Helsinki, and the research protocol was established and published in advance [34].

### 2.2. Subjects

All adult female or male patients diagnosed with MDD and admitted for treatment to inpatient and outpatient departments of the University hospital in the period between June 2022 and December 2024 were invited to participate in this study. Healthy control group (CG) subject (without history and current diagnosis of mental disorders) recruitment was carried out via advertising by primary care physicians.

Of 150 adult patients with MDD and 70 controls invited to participate in this study, 21 patients and 18 CG subjects declined to participate. Then, based on inclusion and exclusion criteria described in our published protocol [34], 100 MDD patients and 52 CG subjects were included in this study. Of those 100 MDD patients, 3 were excluded due to blood sample haemolysis, 9 due to uninformative MRI (presence of braces, errors, and artifacts in sequence acquisition), and 3 due to post-processing MRI errors and artifacts. Of the 52 controls, 1 was excluded due to post-processing MRI errors and artifacts.

In total, 85 MDD patients and 51 healthy controls were included in the present study (*n* = 136). All included subjects signed written informed consent.

### 2.3. Investigations

#### 2.3.1. Clinical

The demographic data about the study subjects, including age, gender, smoking status, body mass index (BMI), somatic comorbidities, time since initial diagnosis of MDD in years, and duration of current depressive episode in months was collected. The Montgomery–Åsberg Depression Rating Scale (MADRS-SIGMA) questionnaire was used to determine the severity of depressive symptoms [35].

#### 2.3.2. Laboratory

Peripheral venous blood was drawn, centrifuged, stored, and analysed using commercial ELISA kits to measure the levels of peripheral endogenous lipopolysaccharide (LPS) and inflammatory cytokines: interleukin 6 (IL-6), interleukin 10 (IL-10), interferon-γ (IFN-γ), and tumour necrosis factor α (TNF-α), according to the published study protocol [34].

#### 2.3.3. Imaging

##### Acquisition

Brain MRI for all subjects was performed on a single 3T Siemens MAGNETOM Skyra scanner using a 20-channel head coil (Siemens Healthineers, Erlangen, Germany). The scanning protocol included standard sequences—3D T1-weighted MPRAGE (sagittal orientation, isotropic, slice thickness 0,9 mm, TR 1650 ms, TE 2.29 ms, TI 900 ms, flip angle 8°) and 3D T2-weighted dark-fluid (FLAIR) (sagittal orientation, isotropic, slice thickness 1 mm, TR 7000 ms, TE 394 ms, TI 2100 ms, flip angle 120°). The main study sequence was a research-focused modification of ASL—the diffusion-prepared 3D pseudo-continuous arterial spin labelling (DP-pCASL) (University of Southern California, USC Mark and Mary Stevens Neuroimaging and Informatics Institute, Los Angeles, CA, USA) [19,36]. The DP-pCASL voxel size was 3.5 × 3.5 × 8.0 mm (slice thickness being 8.0 mm), TR 4200 ms, TE 36.9 ms, flip angle 120°, FOV = 224 mm, matrix size 64 × 64, 12 slices (with 10% oversampling) [19,37]. Several image acquisitions were performed with post-labelling delay (PLD) values of 900 ms and 1800 ms, and b values of 0, 14, and 50 s/mm^2^, as per the protocol [19,22,37]. The total DP-pCASL acquisition time was 10 min. Because 12 DP-pCASL slices of 8 mm thickness were not sufficient to cover the whole brain, localizer placement was performed in a standardised manner by the same person (S.J.) during each subjects’ scanning. Placement was optimised to encompass as much of the brain as possible (sacrificing a small part of the vertex, occipital lobe, and most of the cerebellum and brainstem) and to include the four pre-selected regions that are analysed in this study—the amygdala, ventromedial prefrontal and dorsolateral prefrontal, and cingulate regions. The regions were chosen based on their availability on the Desikan–Killiany atlas and their already established significant role in various aspects of MDD pathophysiology (Appendix A, Table A1).

##### Processing

**Figure 1:**
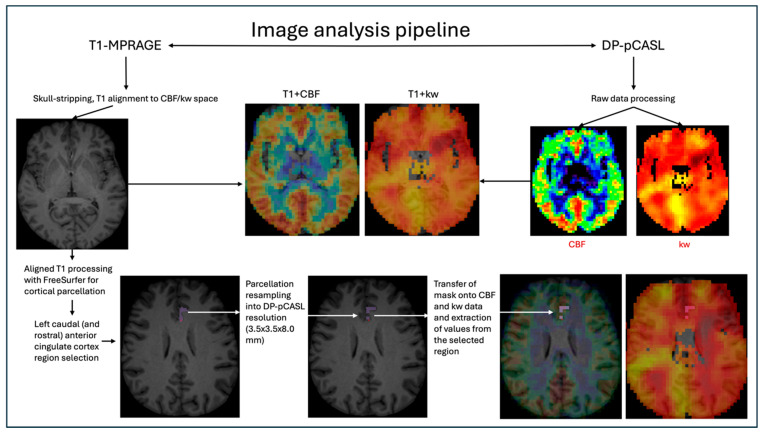
Image analysis pipeline for global and regional kw and CBF analysis. DP-pCASL—diffusion-prepared pseudo-continuous arterial spin labelling; CBF—cerebral blood flow; kw—blood–brain barrier permeability to water. Global CBF and kw maps acquired after raw data processing, visual assessment, and artifact elimination were used for global analyses. Selected regional CBF and kw after ROI selection, parcellation resampling and data extraction were used for regional analyses. Example shown with extraction of kw and CBF from the left caudal anterior cingulate cortex.

Raw DP-pCASL data were exported from the MRI scanner and processed with proprietary BBB water exchange mapping software (University of Southern California, USC Mark and Mary Stevens Neuroimaging and Informatics Institute, Los Angeles, CA, USA) to produce the cerebral blood flow (CBF) and the water exchange rate across the blood–brain barrier (kw) images in nii format [19]. The analysis steps performed by the mapping toolbox included N/2 phase correction, motion correction, co-registration, and quantification of the arterial transit time, CBF, and kw [19]. The images were visually assessed for quality. Some images had an artefactual signal from the skull and orbits which was removed manually using FreeView software (as part of FreeSurfer v.7.4.1). T1-weighted MPRAGE images were inspected visually for motion artifact during scanning and before further image processing. T1-weighted MPRAGE images were skull stripped using SynthStrip software (https://surfer.nmr.mgh.harvard.edu/docs/synthstrip/, accessed 15 November 2025) [38] and registered to DP-pCASL space using AFNI (version 23.2.12). Registration quality was assessed visually; in cases of suboptimal alignment, corrections were made manually until the best possible fit was achieved. DP-pCASL-aligned T1-weighted MPRAGE images were analysed using the FreeSurfer (version 7.4.1) recon-all script. Cortical parcellation and subcortical segmentation volumes were resampled into DP-pCASL resolution (3.5 × 3.5 × 8.0 mm). FreeSurfer-derived segmentation volumes were transferred onto CBF and kw maps, and were used to extract the mean CBF and kw values from relevant regions of interest, which on the FreeSurfer Desikan–Killiany atlas corresponded to the amygdala, the medial orbitofrontal cortex (representing ventromedial prefrontal cortex), the rostral middle frontal cortex (representing dorsolateral prefrontal cortex), and all of the cingulate cortical regions—rostral and caudal anterior, posterior, and isthmus [39]; there were 7 regions per hemisphere, 14 in total. The image processing pipeline is summarised in Figure 1.

### 2.4. Statistical Analyses

Statistical analyses were conducted using IBM SPSS version 30.0.0.0 (172) (IBM Corporation) and JASP version 0.19.3. Data normality was assessed employing the Shapiro–Wilk test. Continuous variables were compared between the MDD and control groups using independent sample *t*-tests or Mann–Whitney U tests, as appropriate. Categorical variables, such as gender, smoking status, and presence of somatic comorbidities, were compared using the chi-squared (χ^2^) test.

Correlations between continuous variables were assessed using Pearson’s or Spearman’s correlation coefficients depending on the normality of distribution. In regional analyses, false discovery rate (FDR) correction was used to reduce the likelihood of a false discovery rate (for 14 comparisons, based on the number of studied regions) [40].

Logistic regression was used to estimate the odds ratios (ORs) and to adjust for confounding variables. Several continuous predictors, such as BMI, IL-10, LPS, and CBF, were dichotomised based on the optimal cut-off value determined using a receiver operating curve (ROC) analysis for the best discriminatory performance between individuals with MDD and the control group. Cut-offs chosen for dichotomised IL-10 and LPS variables (LPS > 143.58 pg/mL and IL-10 > 0 pg/mL) were derived from our previous study of the same cohort (currently in peer review).

## 3. Results

### 3.1. Sociodemographic, Clinical Characteristics and Inflammatory Markers

The sociodemographic and clinical characteristics, LPS, and cytokine concentrations are summarised in Table 1. Comorbidities present in subjects included cardiovascular (arterial hypertension, dyslipidaemia and thyroid disease, diabetes, and asthma) were stable and/or well controlled. There were significantly more smokers in the MDD group when compared with controls (40% and 12%, respectively; χ^2^ = 12.24, *p* < 0.001).

LPS, IL-10, and IFN-γ concentrations were significantly higher in the MDD group compared with the CG (LPS medians of 148 pg/mL and 97 pg/mL, respectively; IL-10 medians of 1.74 pg/mL and 0 pg/mL, respectively; IFN-γ medians of 3.28 pg/mL and 1.30 pg/mL, respectively; all *p* < 0.001). LPS > 143.58 pg/mL and/or IL-10 > 0 pg/mL was present in 94.1% of the MDD group but only 35.3% of the control group.

### 3.2. Global Cerebral Blood Flow and Blood–Brain Barrier Water Exchange

Comparisons of the global kw and CBF are summarised in Table 2. The results did not show significant global kw differences between the MDD group and the CG. However, the global CBF was found to be significantly lower in the MDD group compared with the CG (means of 51.10 mL/100 g/min vs. 57.21 mL/100 g/min, respectively; t = 2.781, *p* = 0.006). When separated by gender, the difference did not reach statistical significance for females (means of 54.53 mL/100 g/min and 58.91 mL/100 g/min, respectively; t = 1.795, *p* = 0.076) but remained significant for males (means of 41.28 mL/100 g/min and 51.68 mL/100 g/min, respectively; t = 3.036, *p* = 0.005).

### 3.3. Regional Cerebral Blood Flow and Blood–Brain Barrier Water Exchange

Comparisons of the regional kw and CBF are summarised in Table 3. Similarly to global kw, there were no significant differences in the kw in any of the 14 studied regions between the study groups. However, the mean regional CBF was significantly lower in patients with MDD when compared with CG in the bilateral medial orbitofrontal cortices, rostral middle frontal cortices, rostral anterior cingulate cortices, posterior cingulate cortices, and the right caudal anterior cingulate cortex.

### 3.4. Logistic Regression Modelling of MDD Predictors

The multivariate logistic regression modelling of independent predictors of MDD is presented in Table 4. Covariates controlling for possible confounders included age, gender, BMI, smoking status, and somatic comorbidities. Regional CBF data were included in the model by averaging the mean CBF of the nine regions that differed significantly between groups (Table 3). In the ROC analysis, a composite regional CBF of less than 59.22 mL/100 mg/min had a Youden’s index of 0.345, a moderate sensitivity of 65.9%, a moderate specificity of 68.6%, and an AUC of 0.679.

The model presented in Table 4 had a good fit (Nagelkerke R Square coefficient 0.557, Hosmer–Lemeshow test χ^2^ = 0.863, *p* = 0.353, maximum VIF 1.485, minimum tolerance 0.673). The model had an overall accuracy of 83.1%, an AUC of 0.876, a sensitivity of 94.1%, a specificity of 64.7%, a positive predictive value of 81.6%, and a negative predictive value of 86.8%. Significant independent predictors of MDD were LPS > 143.58 pg/mL and/or IL-10 > 0 pg/mL (B = 3.358, *p* < 0.001, OR 28.744) and a composite regional CBF (B = 1.760, *p* = 0.004, OR 5.813), while all other variables were not significant.

### 3.5. Associations of kw and CBF with Sociodemographic, Clinical Characteristics, and Inflammatory Marker Concentrations Among MDD Patients

Despite there being no differences in the global kw between the MDD and control groups, among patients with MDD, the global kw was weakly positively associated with the total MADRS score (r = 0.231, *p* = 0.034). The multivariate linear regression model with age, gender, smoking status, BMI, and global kw as covariates was weak (R^2^ = 0.105); however, the global kw was the only significant independent predictor of the MADRS score (B = 0.105, 95% CI for B 0.009–0.202, *p* = 0.033) in the model.

Global CBF was not associated with the total MADRS score (r = 0.094, *p* = 0.392) or global kw (r = 0.196, *p* = 0.072). Global CBF was negatively correlated with age (ρ = −0.379, *p* < 0.001), with similar correlations present in all studied regions except for the amygdala. Global CBF was weakly negatively correlated with BMI (ρ = −0.217, *p* = 0.047); however, when looking at regional correlations, multiple negative regional CBF correlations with BMI did not survive multiple comparison correction. Global kw was not correlated with age (ρ = −0.196, *p* = 0.072). Global and regional kw and CBF were not associated with MDD illness or episode duration, or with peripheral concentrations of LPS, IL-6, IL-10, TNF-α, or IFN-γ, and did not differ between groups divided by smoking status or the presence of somatic comorbidities.

## 4. Discussion

In this study, we found that the global and regional CBF was lower in patients with MDD; however, no such differences were found for the BBB permeability to water. This clear difference answers our main question of which parameter—CBF or BBB water exchange—is more altered in MDD. By using one MRI sequence to acquire both parameters, we could eliminate possible variability in acquisition, sampling, and post-processing. Neither the CBF nor the BBB water exchange were associated with LPS or peripheral inflammatory markers.

The absence of significant differences and associations in the water exchange rate data is somewhat surprising. Studies using other imaging methods showed altered (either increased or decreased) BBB permeability to be associated with MDD or depressive symptoms in other pathologies [10,11,15,16,41]. The absence of water exchange differences specifically in MDD could have several interpretations.

First, it is possible for an MDD-associated BBB disruption to occur via other mechanisms, by changing the effect of membrane carrier proteins other than AQP4. However, if MDD is associated with TJ disruption, the water exchange should also be affected, given that water molecules are small and able to pass paracellularly without intact TJs. The altered expression of AQP4 channels in the presence of other types of BBB disruption could hypothetically act to stabilise the BBB water exchange to more normal levels.

Second, the effect on the water exchange could be too small or too localised to be detected via DP-pCASL imaging, which notably has a low spatial resolution. However, our sample size was greater than some studies employing this same method in other populations which found significant differences in the kw [19,20,21,24,42]. This suggests that the BBB water exchange may not be a prominent mechanism of BBB disruption in patients with MDD.

Additionally, a study comparing DP-pCASL and multi-echo ASL (ME-ASL) found that the two approaches resulted in significantly different measured water exchange values, suggesting the possibility that using other methods of water exchange imaging may reveal associations that were possibly missed using the current approach [43]. Indeed, both of our groups had a mean kw of 128 min^−1^, which is comparable but slightly higher than the kw measured by DP-pCASL in the mentioned study (105 min^−1^), and far from the kw value the authors measured by ME-ASL (301.5 min^−1^) [43]. The application of the diffusion-weighted imaging of free water may further elaborate on the role of water exchange in MDD, as it was shown that white matter free water mediates the association between BBB permeability to water and executive function in older adults [44].

Furthermore, all our participants were not drug-naïve, having recently taken or currently taking antidepressants. There is data in the literature suggesting that antidepressants can regulate BBB permeability [45]. Further studies with DP-pCASL imaging should be conducted to study and compare the water exchange rate in drug-naïve and drug-taking MDD patients.

Finally, while differences were not apparent globally or in studied regions, we did not perform a brain-wide association study. We focused our neuroradiological investigation on a few pre-selected regions (amygdala, ventromedial and dorsolateral prefrontal, and cingulate cortices) which are known to be prominently involved in MDD pathophysiology (Appendix A, Table A1). We opted for this more hypothesis-driven approach rather than exploring brain-wide associations due to concerns in recent years about the problem of reproducibility and false positives in such wide scope association investigations and a subsequently suggested option of performing more focused studies [46,47]. By selecting the regions to be studied before data collection began, we aimed to reduce the possibility of bias and the reporting of associations inflated by chance in our modest sample size. The absence of water exchange differences in the studied regions, which are prominently involved in processes relevant to MDD pathophysiology, remains an important finding, especially given that CBF alterations were clearly demonstrated using the same MRI sequence. The selected regions (specifically, the dorsal anterior cingulate and the dorsolateral prefrontal regions) will be studied in greater detail with additional MRI modalities (including spectroscopy) in later analyses to form a broader multiparametric understanding of their role in MDD [34].

The global water exchange rate was weakly positively correlated with the MADRS scores in MDD patients, suggesting that increased permeability to water may be associated with more pronounced depressive symptoms. A DCE-MRI study of 36 bipolar patients and 14 controls also found that a subgroup of bipolar patients with a globally higher BBB permeability exhibited higher depression symptom severity, also measured by the MADRS [41].

The finding of globally decreased CBF in MDD is consistent with the previous research, summarised in a meta-analysis of 15 previous studies [25]. The global CBF was significantly negatively correlated with age in the MDD group, and age-related decreases in the CBF are well documented in the literature [48]. Notably, the global CBF was not associated with MDD duration, suggesting that age rather than length of the disease is the more important time parameter for CBF. The global CBF in women was significantly higher when compared with men in both groups, also consistent with the previous findings [49,50]. Dehydroepiandrosterone sulfate (DHEAS), which is present in higher concentrations in men and is associated with a lower CBF, has been shown to be an important mediating factor for the difference in CBF between men and women, as well as for mediating the link between gender and depression symptom severity in patients with MDD [49,51].

In patients with MDD, the regional CBF was decreased in all studied ROIs except for the amygdala and isthmic cingulate cortex—including the medial orbitofrontal (ventromedial), the rostral middle frontal (dorsolateral) prefrontal regions, and all other cingulate regions. The unchanged CBF in the amygdala in our study is not consistent with a report of increased right-sided amygdalar CBF in MDD; however, it was one of the two regions which did not show a reduced CBF, suggesting the need for further clarification of the role of the amygdalar CBF in MDD [52].

Logistic regression modelling showed that a combination of LPS, IL-10, and CBF could distinguish MDD patients from controls with an accuracy of 83.1%. LPS and IL-10 were stronger predictors than the CBF. The CBF alone had a moderate sensitivity and specificity (65–68%), which was slightly lower than in the published literature using similar ASL techniques (75–80%) [53]. There were no correlations between the studied peripheral inflammatory markers and the CBF, suggesting the independent roles of LPS, IL-10, and CBF in MDD pathogenesis.

The finding that both inflammatory markers and CBF were independently altered in MDD may help inform further studies and therapeutic development by focusing on the pathophysiological mechanisms involving both processes.

We have found no differences between IL-6 levels between the MDD group and the healthy controls, which contrasts with the findings from other studies [54]. The absence of group differences may reflect the heterogeneity of MDD. Inflammatory alterations appear to characterise only a subset of depressive patients, and our sample may not have included those individuals with elevated IL-6. Moreover, increased IL-6 has been more consistently reported in patients with greater depressive severity and treatment-resistant depression, whereas our MDD group showed moderate depressive symptoms [55,56]. Sex-specific effects with IL-6 differences have also been reported but were beyond the scope of the present analyses [57]. Additionally, most MDD group individuals (97.6%) in our sample were receiving antidepressant treatment, which has been shown to reduce IL-6 levels in a meta-analysis [58]. Importantly, IL-6 alterations may be more pronounced in specific depressive subtypes, such as atypical and melancholic depression compared with healthy controls [59,60].

Our study has several limitations. Sample sizes were relatively small, which led us to choose to perform a global and focused regional rather than brain-wide association analysis. There were significantly more females than males in our study groups due to the composition of our studied population, therefore we could not perform extensive comparisons between females and males. Also, all patients with MDD were taking (or have recently been taking) antidepressant medication, which can potentially influence our studied parameters (particularly the water exchange rate). Regional analyses may have suffered from low DP-pCASL resolution in comparison to typical MRI sequences, with possible volume averaging with nearby regions (especially the adjacent white matter). Also, region selection was limited by the parcellation of the Desikan–Killiany atlas. In some cases, T1 registration to DP-pCASL space was suboptimal, and while manual corrections were made to achieve the best possible fit, small inconsistencies may have remained (such as the DP-pCASL signal extending into the ventricles or beyond the cortical border), leading to the T1-derived regions of interest not aligning perfectly with the regions on lower resolution DP-pCASL data. Finally, in the literature, DP-pCASL imaging was found to produce significantly different kw values when compared with ME-ASL imaging, suggesting that our results may need to be validated with other methods of water exchange imaging.

## 5. Conclusions

DP-pCASL imaging confirmed the previous findings of global and regional CBF reduction in MDD patients but showed no differences in the BBB water exchange compared to controls. This suggests the greater significance of the CBF than the BBB water exchange in the pathophysiology of MDD. A possible weak association between the global water exchange and depression symptom severity in MDD patients may suggest that increased water permeability is associated with more severe symptoms in established MDD, which warrants confirmation and further investigation. CBF, LPS, and IL-10 were found to be significant independent predictors of MDD.

## Figures and Tables

**Table 1:** Sociodemographic and clinical characteristics, LPS and cytokine concentrations; N = 136.

	MDD, *n* = 85	Controls, *n* = 51	Group Differences
Age, years; median (IQR)	42.0 (26.0)	39.0 (24.0)	U = 2344.5, *p* = 0.426
Gender			χ^2^ = 0.094, *p* = 0.759
Female, *n* (%)	63 (74.1)	39 (76.5)	
Male, *n* (%)	22 (25.9)	12 (23.5)	
Body mass index, kg/m^2^; median (IQR)	24.51 (7.78)	24.67 (8.19)	U = 2256.0, *p* = 0.691
Smoking status			**χ^2^ = 12.24, *p* < 0.001**
Yes, *n* (%)	34 (40)	6 (11.8)	
No, *n* (%)	51 (60)	45 (88.2)	
Somatic comorbidities			χ^2^ = 0.863, *p* = 0.353
Yes, *n* (%)	13 (15.3)	11 (21.6)	
No, *n* (%)	72 (84.7)	40 (78.4)	
MDD duration, years; median (IQR)	8 (11)	-	-
Current MDD episode duration, months; median (IQR)	5 (8)	-	-
MADRS, total score; median (IQR)	30 (11)	2 (3)	**U = 4331.0, *p* < 0.001**
Use of psychotropic medications, *n* (%)	85 (100)	0	-
Antidepressant, *n* (%)	83 (97.6)	0	
Benzodiazepine, *n* (%)	57 (67.1)	0	
Low-dose antipsychotic, *n* (%)	50 (58.8)	0	
Mood stabiliser, *n* (%)	7 (8.2)	0	
LPS, pg/mL; median (IQR)	148.04 (163.73)	97.15 (76.57)	**U = 2940.5, *p* < 0.001**
IL-6, pg/mL; median (IQR)	1.36 (2.63)	1.34 (2.53)	U = 2201.0, *p* = 0.880
IL-10, pg/mL; median (IQR)	1.74 (2.85)	0 (0)	**U = 3489.0, *p* < 0.001**
TNFα, pg/mL; median (IQR)	0 (0)	0 (0)	U = 2295.0, *p* = 0.079
IFN-γ, pg/mL; median (IQR)	3.28 (5.96)	1.30 (3.90)	**U = 2898.0, *p* < 0.001**
LPS > 143.58 pg/mL and/or IL-10 > 0 pg/mL			**χ^2^ = 57.780, *p* < 0.001**
Yes, *n* (%)	80 (94.1)	18 (35.3)	
No, *n* (%)	5 (5.9)	33 (64.7)	

MDD—major depressive disorder; MADRS—Montgomery–Åsberg Depression Rating Scale; LPS—lipopolysaccharide; IL-6—interleukin 6; IL-10—interleukin 10; TNFα—tumour necrosis factor; IFN-γ—interferon gamma; U—Mann–Whitney U test statistic; χ^2^–chi-squared test statistic; IQR—interquartile range (Q3-Q1). Statistically significant differences marked in bold.

**Table 2:** Comparison of global kw and CBF; N = 136.

	MDD, *n* = 85	Controls, *n* = 51	Group Differences
kw, min^−1^; mean (SD)	127.97 (17.89)	128.14 (19.50)	t = 0.053, *p* = 0.958
female	128.96 (16.64)	128.43 (16.86)	t = −0.155, *p* = 0.877
male	125.14 (21.25)	127.22 (27.29)	t = 0.246, *p* = 0.807
CBF, mL/100 g/min; mean (SD)	51.10 (13.45)	57.21 (10.39)	**t = 2.781, *p* = 0.006**
female	54.53 (12.73)	58.91 (10.62)	t = 1.795, *p* = 0.076
male	41.28 (10.43)	51.68 (7.56)	**t = 3.036, *p* = 0.005**

MDD—major depressive disorder; kw—water exchange rate across the blood–brain barrier; CBF—cerebral blood flow; SD—standard deviation; t—Student’s T test statistic. Statistically significant differences marked in bold.

**Table 3:** Comparison of regional kw and CBF; N = 136.

	MDD, *n* = 85	Controls, *n* = 51	Group Differences
Amygdala			
Left			
kw, min^−1^; mean (SD)	121.56 (20.42)	124.01 (22.98)	t = 0.645, *p* = 0.520
CBF, mL/100 g/min; mean (SD)	44.17 (10.31)	46.81 (10.00)	t = 1.460, *p* = 0.147
Right			
kw, min^−1^; mean (SD)	120.92 (23.94)	112.13 (24.82)	t = −2.044, *p* = 0.043
CBF, mL/100 g/min; median (IQR)	43.42 (12.59)	46.29 (13.29)	U = 1749.0, *p* = 0.060
Medial orbitofrontal cortex (MOC)			
Left			
kw, min^−1^; mean (SD)	125.39 (19.64)	125.04 (22.13)	t = −0.097, *p* = 0.923
CBF, mL/100 g/min; mean (SD)	50.36 (13.60)	57.75 (11.17)	**t = 3.272, *p* = 0.001**
Right			
kw, min^−1^; mean (SD)	130.02 (19.65)	125.94 (23.87)	t = −1.080, *p* = 0.282
CBF, mL/100 g/min; mean (SD)	52.03 (14.33)	58.59 (10.91)	**t = 2.818, *p* = 0.003**
Rostral middle frontal cortex (RMFC)			
Left			
kw, min^−1^; median (IQR)	133.39 (33.11)	135.12 (29.38)	U = 2088.0, *p* = 0.721
CBF, mL/100 g/min; mean (SD)	51.48 (15.98)	61.47 (13.37)	**t = 3.747, *p* < 0.001**
Right			
kw, min^−1^; mean (SD)	129.51 (21.27)	130.67 (20.51)	t = 0.311, *p* = 0.756
CBF, mL/100 g/min; mean (SD)	52.90 (15.67)	60.75 (13.46)	**t = 2.980, *p* = 0.003**
Rostral anterior cingulate cortex (rACC)			
Left			
kw, min^−1^; mean (SD)	134.23 (22.05)	132.27 (22.91)	t = −0.495, *p* = 0.621
CBF, mL/100 g/min; mean (SD)	65.04 (16.91)	74.15 (13.43)	**t = 3.467, *p* < 0.001**
Right			
kw, min^−1^; mean (SD)	138.69 (24.27)	133.89 (24.93)	t = −1.113, *p* = 0.268
CBF, mL/100 g/min; mean (SD)	68.48 (17.71)	75.34 (14.51)	**t = 2.335, *p* = 0.021**
Caudal anterior cingulate cortex (cACC)			
Left			
kw, min^−1^; mean (SD)	112.30 (28.17)	109.72 (28.57)	t = −0.515, *p* = 0.608
CBF, mL/100 g/min; mean (SD)	47.27 (17.88)	53.21 (15.26)	t = 1.979, *p* = 0.050
Right			
kw, min^−1^; mean (SD)	115.27 (24.05)	119.51 (24.27)	t = 0.992, *p* = 0.323
CBF, mL/100 g/min; mean (SD)	48.49 (16.29)	57.29 (15.64)	**t = 3.097, *p* = 0.002**
Posterior cingulate cortex (PCC)			
Left			
kw, min^−1^; mean (SD)	101.38 (30.44)	107.96 (27.55)	t = 1.263, *p* = 0.209
CBF, mL/100 g/min; mean (SD)	48.56 (16.77)	57.48 (17.97)	**t = 2.925, *p* = 0.004**
Right			
kw, min^−1^; mean (SD)	105.22 (27.82)	108.33 (30.07)	t = 0.611, *p* = 0.542
CBF, mL/100 g/min; mean (SD)	50.92 (17.06)	58.17 (19.28)	**t = 2.283, *p* = 0.024**
Isthmus cingulate cortex (ICC)			
Left			
kw, min^−1^; mean (SD)	120.34 (29.30)	128.79 (29.60)	t = 1.622, *p* = 0.107
CBF, mL/100 g/min; mean (SD)	63.44 (18.54)	69.27 (16.76)	t = 1.838, *p* = 0.068
Right			
kw, min^−1^; mean (SD)	120.70 (28.94)	125.73 (30.01)	t = 0.968, *p* = 0.335
CBF, mL/100 g/min; mean (SD)	63.39 (19.28)	69.81 (18.84)	t = 1.897, *p* = 0.060

MDD—major depressive disorder; t—independent samples T-test statistic; U—Mann–Whitney U test statistic; CBF—cerebral blood flow; kw—water exchange rate across the blood–brain barrier; SD—standard deviation; IQR—interquartile range (Q3–Q1). Statistically significant differences after false discovery rate (FDR) correction marked in bold.

**Table 4:** Multivariate logistic regression of independent predictors of MDD, N = 136.

Model		Coefficient B	*p* Value	OR (95% CI)
Factors	Age, years	0.000	0.994	1.000 (0.996–1.041)
	Male gender	−0.551	0.384	0.577 (0.167–1.994)
	BMI > 30 kg/m^2^	0.540	0.455	1.716 (0.416–7.078)
	Smoking status	0.614	0.297	1.848 (0.583–5.860)
	Somatic comorbidities	−0.131	0.846	0.878 (0.236–3.267)
	LPS > 143.58 pg/mL and/or IL-10 > 0 pg/mL	3.358	**<0.001**	28.744 (8.387–98.503)
	Composite regional CBF < 59.22 mL/100 g/min	1.760	**0.004**	5.813 (1.782–18.969)

MDD—major depressive disorder; BMI—body mass index; LPS—lipopolysaccharide; IL-10—interleukin 10; CBF—cerebral blood flow; OR—odds ratio; CI—confidence interval. Composite regional CBF was calculated by averaging the CBF of the bilateral rostral middle frontal, medial orbitofrontal, rostral anterior and posterior cingulate cortices, and the right caudal anterior cingulate cortex. Statistically significant *p* values marked in bold.

## Data Availability

The data supporting the conclusions of this article will be made available by the authors on reasonable request due to technical and privacy reasons.

## Abbreviations

The following abbreviations are used in this manuscript:

MRI	Magnetic resonance imaging
DP-pCASL	Diffusion-prepared pseudo-continuous arterial spin labelling
CBF	Cerebral blood flow
BBB	Blood–brain barrier
kw	Water exchange across the blood–brain barrier
MDD	Major depressive disorder
LPS	Lipopolysaccharide
IL-6	Interleukin 6
IL-10	Interleukin 10
TNF-α	Tumour necrosis factor α
IFN-γ	Interferon-γ
TJ	Tight junction
AQP4	Aquaporin 4
GBCA	Gadolinium-based contrast agent
DCE-MRI	Dynamic contrast-enhanced magnetic resonance imaging
PET	Positron emission tomography
ASL	Arterial spin labelling
CSF	Cerebrospinal fluid
MADRS	Montgomery–Åsberg Depression Rating Scale
FDR	False discovery rate
BMI	Body mass index
ROC	Receiver operating curve
CG	Control group
IQR	Interquartile range
SD	Standard deviation
MOC	Medial orbitofrontal cortex
RMCF	Rostral middle frontal cortex
rACC	Rostral anterior cingulate cortex
cACC	Caudal anterior cingulate cortex
PCC	Posterior cingulate cortex
ICC	Isthmus cingulate cortex
AUC	Area under curve

## Appendix A

The description of the role of selected studied regions in MDD pathophysiology is presented in Table A1.

**Table A1:** Pre-selected regions for analysis and some of their described changes in the pathophysiology of major depressive disorder.

Region	Significance in MDD	Study Protocol	Reference
Amygdala	Increased activity in automatic negative stimuli processing	fMRI study of 57 patients with MDD and 37 controls	[61]
	Reduced intrinsic connectivity	fMRI study of 55 patients with MDD and 19 controls	[62]
	Unpredictable chronic mild stress associated with increased inflammatory cytokines (IL-1β, IL6, TNF-α)	Mouse model with experimental and control groups, messenger RNA quantification in the amygdala	[63]
	Increased activity in young adults with family history of depression, possible marker of low resilience	fMRI study of 27 young adults with family history of MDD and 45 controls without family history	[64]
	Increased CBF of the right amygdala increases risk of MDD in first-degree relatives of patients with MDD	ASL MRI 33-month follow-up study of 200 first-degree relatives of MDD patients and 100 controls with no first-degree family history of MDD	[52]
vmPFC	Ventromedial frontal cortex hypoactivity during reward positivity and correlation with anhedonia in patients with MDD	MEG study of 43 patients with MDD	[65]
	vmPFC hyperactivity predicts poor outcomes to sertraline treatment in late-life MDD	fMRI study of 36 older patients with MDD	[66]
	Abnormal vmPFC–amygdala connectivity to happy faces in females with MDD	fMRI study of 19 female patients with MDD during depressive episode and 19 controls	[67]
	Increased cortical thickness of the vmPFC in medication-free patients with MDD	Meta-analysis of 15 structural MRI studies with 529 MDD patients and 586 controls	[68]
DLPFC	Impaired DLPFC neuroplasticity in patients with treatment-resistant MDD	TMS–EEG study of 60 patients with treatment-resistant MDD and 60 controls	[69]
	Normalization of altered DLPFC–precuneus connectivity reduces depressive symptoms	fMRI functional connectivity neurofeedback study of 20 participants	[70]
	20-Hz rTMS over the left DLPFC positively impacted depression severity	rTMS study of 28 female subjects with MDD separated into experimental and control groups	[71]
	Left DLPFC increased functional measures in response to treatment of MDD	Meta-analysis of 37 eligible functional neuroimaging studies	[72]
	tDCS of the DLPFC reduced anhedonia in depressed patients	Randomised double-blind sham-controlled trial of tDCS with 70 patients separated into three groups	[73]
Cingulate	Gray matter volume of the anterior cingulate cortex associated with suicidal ideation in patients with MDD	Meta-analysis of 246 patients with MDD and 235 controls	[74]
	Dysconnectivity of dorsal anterior cingulate cortex with cognition-related regions	Meta-analysis of 44 resting state fMRI studies	[75]
	Increased amplitude of low-frequency fluctuation in the dorsal anterior cingulate cortex in patients with MDD	Resting state fMRI study of 30 first-episode drug-naïve patients with MDD and 52 controls	[76]
	Altered subregional anterior cingulate cortex functional connectivity in patients with MDD and associated with depressive and anxious symptom severity	fMRI study of 41 newly diagnosed drug-naïve patients with MDD and 43 controls	[77]

MDD—major depressive disorder; vmPFC—ventromedial prefrontal cortex; DLPFC—dorsolateral prefrontal cortex; fMRI—functional magnetic resonance imaging; TMS–EEG—transcranial magnetic stimulation–electroencephalography; rTMS—repetitive transcranial magnetic stimulation; tDCS—transcranial direct current stimulation; MEG—magnetoencephalography. Analyses in our study were performed on regions derived and named based on the Desikan–Killiany atlas which include all or most of the pre-selected regions described in the table as follows: amygdala—amygdala; vmPFC—medial orbitofrontal cortex, rostral anterior cingulate cortex; DLPFC—rostral middle frontal cortex; cingulate—rostral and caudal anterior, posterior, isthmic cingulate cortices.
